# Continuing Exposure to Low-Dose Nonylphenol Aggravates Adenine-Induced Chronic Renal Dysfunction and Role of Rosuvastatin Therapy

**DOI:** 10.1186/1479-5876-10-147

**Published:** 2012-07-19

**Authors:** Chia-Hung Yen, Cheuk-Kwan Sun, Steve Leu, Christopher Glenn Wallace, Yu-Chun Lin, Li-Teh Chang, Yung-Lung Chen, Tzu-Hsien Tsa, Ying-Hsien Kao, Pei-Lin Shao, Chi-Ying Hsieh, Yen-Ta Chen, Hon-Kan Yip

**Affiliations:** 1 Department of Biological Science and Technology, National Pingtung University of Science andTechnology, Pingtung, Taiwan; 2 Department of Emergency Medicine, E-DA Hospital, I-Shou University, Kaohsiung, Taiwan; 3 Center for Translational Research in Biomedical Sciences, Kaohsiung Chang Gung Memorial Hospital and Chang Gung University College of Medicine, Kaohsiung, Taiwan; 4 Department of Plastic Surgery, University Hospital of South Manchester, Manchester, UK; 5 Department of Medical Research, E-DA Hospital, I-Shou University, Kaohsiung, Taiwan; 6 Basic Science, Nursing Department, Meiho University, Pingtung, Taiwan; 7 Division of Cardiology, Department of Internal Medicine, Kaohsiung Chang Gung Memorial Hospital and Chang Gung University College of Medicine, Kaohsiung, Taiwan; 8 Department of Materials Science and Engineering, National Cheng Kung University, Tainan, Taiwan; 9 Department of Environmental Science and Engineering, National Pingtung University of Science and Technology, Pingtung, Taiwan; 10 Division of Urology, Department of Surgery, Kaohsiung Chang Gung Memorial Hospital, Kaohsiung, Taiwan

## Abstract

**Background:**

Nonylphenol (NP), an environmental organic compound, has been demonstrated to enhance reactive-oxygen species (ROS) synthesis. Chronic exposure to low-dose adenine (AD) has been reported to induce chronic kidney disease (CKD).

**Methods:**

In this study, we tested the hypothesis that chronic exposure to NP will aggravate AD-induced CKD through increasing generations of inflammation, ROS, and apoptosis that could be attenuated by rosuvastatin. Fifty male Wistar rats were equally divided into group 1 (control), group 2 (AD in fodder at a concentration of 0.25%), group 3 (NP: 2 mg/kg/day), group 4 (combined AD & NP), and group 5 (AD-NP + rosuvastatin: 20 mg/kg/day). Treatment was continued for 24 weeks for all animals before being sacrificed.

**Results:**

By the end of 24 weeks, serum blood urea nitrogen (BUN) and creatinine levels were increased in group 4 than in groups 1–3, but significantly reduced in group 5 as compared with group 4 (all p < 0.05). Histopathology scorings of renal-parenchymal and tubular damages were significantly higher in group 4 than in groups 1–3, but remarkably lower in group 5 compared with group 4 (all p < 0.01). Both gene and protein levels of inflammation, oxidative stress, ROS, and cellular apoptosis were remarkably higher in group 4 compared with groups 1–3, but lowered in group 5 than in group 4 (all p < 0.001). Conversely, both gene and protein levels of anti-oxidants, anti-inflammation and anti-apoptosis were markedly increased in group 5 compared with group 4 (all p < 0.001).

**Conclusion:**

NP worsened AD-induced CKD that could be reversed by rosuvastatin therapy.

## Background

Chronic kidney disease (CKD) has become a growing epidemic that not only puts a substantial burden on global healthcare resources [1-5], but is also an important cause of mortality and disability worldwide [6,7]. The risk factors of CKD have been extensively investigated in many previous epidemiologic and clinical observational studies [1,3-5,8-11]. Although age, diabetes mellitus, and hypertension have been well reported to be the prevalent CKD risk factors [1,11-13], other risk factors including genetic, racial or familial predisposition [14,15], “western” lifestyle [1], infectious diseases with infection-related glomerulonephritis [16], and chronic exposure to certain chemicals [17,18], may also play an important role in the initiation and propagation of CKD. However, the potential impact of chronic exposure to environmental contaminants on the initiation and aggregation of CKD has scarcely been reported.

Nonylphenol (NP), an environmental organic compound derived from alkylphenols, has a chemical structure mimicking that of natural estrogen in the animal/human body [19-21]. Since previous studies have reported that NP interferes with the development and function of endocrine-reproductive systems through binding to the estrogen receptors [19-23], NP and other environmental contaminants have been recently categorized into endocrine-disrupting chemicals [24]. Interestingly, a study using animal model has revealed that chronic exposure to low-dose of NP can induce polycystic kidney disease in rats [25]. The underlying mechanisms, however, has not been fully investigated. Besides, previously animal model study has shown that chronic exposure to adenine (AD), an important component of DNA, can cause crystal formation in kidney parenchyma and also CKD [26,27]. Thus, AD-induced CKD in rats has long been accepted as an experimental model for investigating the pathologies and mechanisms of CKD [26-28].

Although inflammatory reaction, oxidative stress, and the generation of reactive oxygen species (ROS) have been reported to be the principal mechanisms involved in various etiologies of CKD [1,17,18,29-32], whether similar contributors are also responsible for environmental organic compound-induced CKD remains unclear. Beside its action in lowering the serum cholesterol, Rosuvastatin has also been reported to possess anti-inflammatory and anti-oxidant properties [33]. Accordingly, this study tested the hypothesis that chronic exposure to NP will aggravate AD-induced CKD in a rat model that could be attenuated by rosuvastatin therapy. The underlying mechanisms were also investigated.

## Methods

### Ethics

All animal experimental procedures were approved by the Institute of Animal Care and Use Committee at our hospital and performed in accordance with the Guide for the Care and Use of Laboratory Animals (NIH publication No. 85–23, National Academy Press, Washington, DC, USA, revised 1996). All the technicians who performed the bench work blinded in the treatment protocol.

### Animal model and blood sampling for levels of serum creatinine and blood urea nitrogen after CKD induction

Fifty pathogen-free, 8-week-old male Wistar rats, weighing about 250 g (Charles River Technology, BioLASCO Taiwan Co., Ltd., Taiwan) were equally divided into group 1 (control), group 2 [AD in fodder at a concentration of 0.25% (i.e. 1 gm of AD in 100 gm of chow)], group 3 [NP (2.0 mg/kg/day) by gavage], group 4 (combined AD + NP), and group 5 [combined AD + NP + rosuvastatin (20 mg/kg/day) by gavage]. The dosage of rosuvastatin for the animals was based on our previous study [34] and the dosage of AD for the animals was according to previous reports [27-29] with some modifications. The AD was administered in fodder rather than by gavage due to the pharmacokinetic half life of AD was rather shorter.

AD, NP, and rosuvastatin were given to the animals for totally 24 weeks. The body weight, concentrations of serum creatinine and blood urea nitrogen (BUN) were measured every four weeks during the course of the experiment.

### Blood sampling, collection of 24-hour urine for renal function assessment and collection of kidney tissue

By the end of 24 weeks of treatment, the animals were sacrificed. The kidneys were collected for individual study, while the blood samples were collected for serum levels of BUN and creatinine. Quantification of BUN and creatinine level was performed using standard laboratory equipment at our hospital. For further determining the renal function, the ratio of protein to creatinine which was also utilized was based on our recent report [35].

### Hematoxylin and eosin staining, histopathology scoring and Periodic Acid-Schiff (PAS) staining

Kidney specimens from all animals were fixed in 10% buffered formalin before embedding in paraffin. Tissue was sectioned at 5 μm before being stained with hematoxylin and eosin (H & E) for light microscopic analysis. Histopathology scoring was applied as described previously [36] in a blind fashion. The score was given based on grading of tubular necrosis, loss of brush border, cast formation, and tubular dilatation in 10 randomly chosen, non-overlapping fields (200x) as follows: 0 (none), 1 (≤10%), 2 (11–25%), 3 (26–45%), 4 (46–75%), and 5 (≥76%).

Additionally, to determine the architecture of basement membrane and the structure of renal tubular border, Periodic Acid Schiff (PAS) staining was utilized in the present study.

### Immunofluorescence (IF) and Immunohistochemical (IHC) studies

IF staining was performed for the examinations of CD68 (macrophage surface marker)-positive cells using respective primary antibodies. Additionally, IHC labeling technique was adopted for identifying glutathione reductase (GR)-positive cells using respective primary antibody. Irrelevant antibodies were used as controls in the current study.

Western Blot Analyses for Nuclear Factor (NF)-κB, Intercellular Adhesion Molecule (ICAM)-1, Heme Oxygenase (HO)-1, NAD(P)H Quinone Oxidoreductase (NQO) 1, NADPH oxidase (NOX)-1 and NOX-2 in Kidney.

Equal amounts (10–30 μg) of renal protein extracts were loaded and separated by SDS-PAGE using 8–10% acrylamide gradients. Following electrophoresis, the separated proteins were transferred electrophoretically to a polyvinylidene difluoride (PVDF) membrane (Amersham Biosciences). Nonspecific proteins were blocked by incubating the membrane in blocking buffer (5% nonfat dry milk in T-TBS containing 0.05% Tween 20) overnight. The membranes were incubated with the indicated primary antibodies (NQO- 1, 1: 1000, Abcam; GPx, 1: 2000, Abcam; HO-1, 1: 250, Abcam; ICAM-1, 1: 2000, Abcam; NF-κB, 1: 200, Santa Cruz; Actin 1: 10000, Chemicon; NOX-1, 1:1500, Sigma; NOX-2, 1:500, Sigma) for 1 hour at room temperature. Horseradish peroxidase-conjugated anti-rabbit immunoglobulin IgG (1: 2000, Cell signaling) was used as a second antibody for 1 hour at room temperature. The washing procedure was repeated eight times within one hour.

### Western blot analysis for oxidative stress in kidney

The Oxyblot Oxidized Protein Detection Kit was purchased from Chemicon (S7150). The procedure of 2,4-dinitrophenylhydrazine (DNPH) derivatization was carried out on 6 μg of protein for 15 minutes according to the manufacturer’s instructions. One-dimensional electrophoresis was carried out on 12% SDS/polyacrylamide gel after DNPH derivatization. Proteins were transferred to nitrocellulose membranes which were then incubated in the primary antibody solution (anti-DNP 1: 150) for two hours, followed by incubation with second antibody solution (1:300) for one hour at room temperature. The washing procedure was repeated eight times within 40 minutes.

Immunoreactive bands were visualized by enhanced chemiluminescence (ECL; Amersham Biosciences) which was then exposed to Biomax L film (Kodak). For quantification, ECL signals were digitized using Labwork software (UVP). For oxyblot protein analysis, a standard control was loaded on each gel.

### Protocol for RNA extraction

Lysis/binding buffer (High Pure RNA Tissue Kit, Roche, Germany) 400 μL and an appropriate amount of frozen kidney was added to a nuclease-free 1.5 mL microcentrifuge tube, followed by disruption and homogenization of the tissue by using a rotor-stator homogenizer (Roche). The lysate in the microcentrifuge tube was then centrifuged for two minutes at 13,000 *g*. Only the superficially collected supernatant was utilized for subsequent steps. Absolute ethanol 200 μL was added to the lysate supernatant and mixed well. The entire sample in the upper reservoir was pipetted into a High Pure Filter Tube (Roche) that was placed in the Collection Tube (Roche). This sample was then centrifuged for 30 seconds at 13,000 *g* in a standard tabletop microcentrifuge. After that, the Filter Tube was removed from the Collection Tube and the flowthrough liquid was discarded. For each isolation, 90 μL DNase incubation buffer was pipetted into a sterile 1.5 mL reaction tube, 10 μL DNase I working solution was then added, mixed and incubated for 15 minutes at 25 °C. Wash buffer I 500 μL was then added to the upper reservoir of the filter tube, which was then centrifuged for 15 seconds at 8,000 *g*. The filter tube was removed from the Collection Tube and the flowthrough liquid was then discarded. Wash Buffer II 500 μL was added to the upper reservoir of the Filter Tube, which was then centrifuged for 15 seconds at 8,000 *g* and the flowthrough was discarded. Wash buffer II 300 μL was added to the upper reservoir of the filter tube, which was centrifuged for 2 minutes full-speed at approximately 13,000 *g*. The column was then carefully removed from the collection tube such that the column did not contact the flowthrough to avoid ethanol carryover. The filter tube was then inserted into a 1.5 mL nuclease-free and sterilized microcentrifuge tube. Elution Buffer 100 μL was added to the upper reservoir of the filter tube; the tube assembly was then centrifuged for 1 minute at 8,000 *g* resulting in eluted RNA in the microcentrifuge tube.

### Reverse transcription qPCR analysis

Quantitative reverse transcription-polymerase chain reaction (RT-qPCR) was conducted using LightCycler TaqMan Master (Roche, Germany) in a single capillary tube according to the manufacturer’s guidelines for individual component concentrations as we previously reported [37,38]. Forward and reverse primers were each designed based on individual exons of the target gene sequence to avoid amplifying genomic DNA.

During PCR, the probe was hybridized to its complementary single-strand DNA sequence within the PCR target. As amplification occurred, the probe was degraded due to the exonuclease activity of Taq DNA polymerase, thereby separating the quencher from reporter dye during extension. During the entire amplification cycle, light emission increased exponentially. A positive result was determined by identifying the threshold cycle value at which reporter dye emission appeared above background.

### Statistical analysis

Quantitative data are expressed as means ± SD. Statistical analysis was adequately performed by ANOVA followed by Bonferroni multiple-comparison post hoc test. Statistical analysis was performed using SAS statistical software for Windows version 8.2 (SAS institute, Cary, NC). A probability value <0.05 was considered statistically significant.

## Results

### Serial changes of serum levels of creatinine and blood urea nitrogen and the ratio of urine protein to creatinine at the end of study period

**Figure 1:**
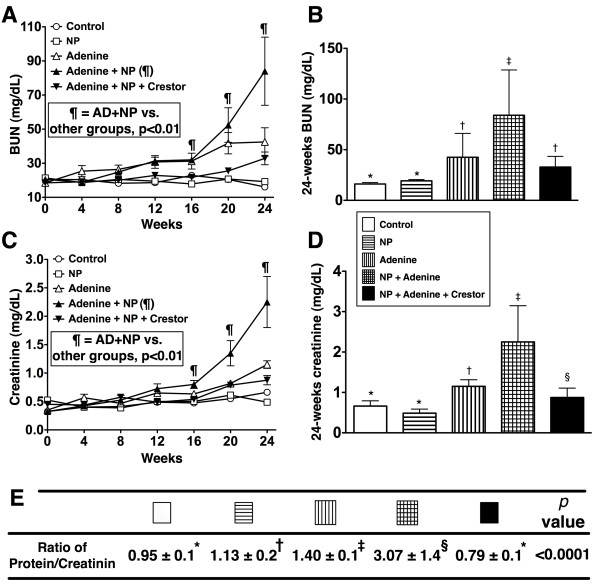
**Serial changes of serum levels of creatinine and blood urea nitrogen and the ratio of urine protein to creatinine at the end of study period.** Serial changes in serum levels of blood urine nitrogen (BUN) (**A**) and creatinine (**C**) in different groups of rats after adenine-induced chronic kidney disease (CKD). B) By 24-week, remarkably increased serum levels of BUN in adenine (AD)-treated (group 3) rats than in normal (group 1) and Nonylphenol (NP)-treated (group **2**) rats. Markedly increased BUN levels in adenine + NP-treated (group 4) rats than in other groups, but significantly reduced in adenine + NP-treated rats receiving rosuvastatin (Rosu) (group 5) than in group 4. D) Similar changes in serum creatinine levels compared to those of BUN by 24-week after CKD induction. Statistical analysis by one-way ANOVA. For BUN and creatinine: * vs. other groups, p < 0.0001 (at 24 week). For A & C): ¶ indicated AD + NP group vs. other groups (at 16, 20 and 24 weeks), p < 0.01. For E) (the ratio of urine protein to creatinine): * vs. other groups with different symbols (†, ‡, §), p < 0.0001. Symbols (*, †, ‡, §) indicate significance (at 0.05 level by Scheffe multiple-comparison post hoc test) (n = 10 in each group).

The serum levels of BUN and creatinine did not differ among the five groups of animals at the beginning and within the first two months of the study period (Figure 1A and C) (p > 0.5). Additionally, the BUN and creatinine levels did not differ between group 1 (normal control) and group 2 (NP only) at the end of the study period (i.e. 24 weeks) (Figure 1B and D) (p > 0.1). However, the BUN and creatinine levels were notably increased in group 3 (AD only) at end of 16 weeks and further increased at the end of the study period as compared with groups 1 and 2 (all p < 0.01). Furthermore, the BUN and creatinine levels were notably increased in group 4 (AD + NP) at the 16 week (all p < 0.005) and more substantially increased at the end of the study period compared with those animals in groups 1, 2 and 3 (Figure 1A and C) (p < 0.0001). Conversely, these two parameters were markedly suppressed in group 5 (AD + NP + rosuvastatin) when compared with group 4 in the end of study period (all p < 0.001). Additionally, the BUN level showed no difference between groups 3 and 5 (p > 0.1), whereas the creatinine level was notably lower in group 5 than in group 3 at the end of study period (p < 0.01). Furthermore, these two parameters were remarkably higher in group 5 than in groups 1 and 2 at the end of study period (Figure 1B and D) (all p < 0.01).

The ratio of urine level of protein to creatinine did not differ between groups 1 and 5. However, the ratio was remarkably higher in groups 2, 3 and 4 than that in groups 1 and 5, notably higher in groups 3 and 4 than that in 2 group, and significantly higher in group 4 than that in group 3, but it showed no difference between groups 1 and 5 (Figure 1E).

### Histopathological scoring of the kidneys

**Figure 2:**
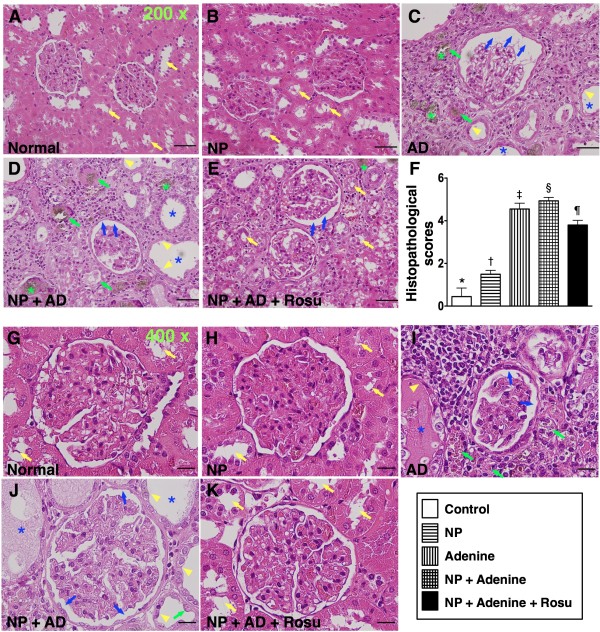
**Histological scores of kidney by 24-week after CKD induction.** H & E staining [200 x (A to E) & 400x (G to K)] of kidney sections in groups 1 to 5 animals (**A to K**) showing notably higher degree of loss of brush border in renal tubules (yellow arrowheads), cast formation (blue asterisk), tubular dilatation (blue arrows), tubular necrosis (green arrows), and less intact of renal tubule (yellow arrows) in group 3. Further enhanced damage in group 4 compared with that in groups 1 and 2, but notably improved in group 5 after rosuvastatin treatment. Also note crystal formations (green asterisk) in groups 3 and 4 animals. **F)** * vs. other groups, p < 0.0001 (by one-way ANOVA). Symbols (*, †, ‡, §, ¶) indicate significance (at 0.05 level by Bonferroni multiple comparison post hoc test) (n = 10 in each group). Scale bars in right lower corner represent 50 μm in **A-E** and 20 μm in **G-K**.

To evaluate the effect of rosuvastatin on NP + AD-induced renal injury, histological scoring based on the typical microscopic features of chronic tubular damage, including extensive tubular necrosis and dilatation, as well as cast formation and loss of brush border was adopted (Figure 2). The injury was found to be more severe in group 4 than in groups 1 to 3, notably more severe in group 3 than in groups 1 and 2, but the degree of injury was less remarkable in group 5 compared with groups 3 and 4, suggesting that rosuvastatin therapy significantly protected the kidney from AD or NP + AD damage (Figure 2).

**Figure 3:**
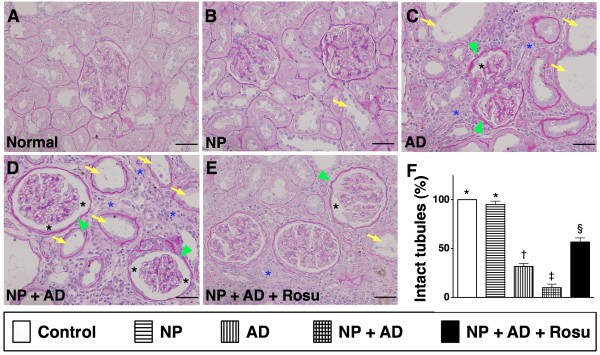
**Percentage of intact renal tubules in CKD rats.** Periodic Acid Schiff (PAS) staining (200 x) of kidney sections in groups 1 to 5 animals (**A, B, C, D, E**) showing notable renal tubular destruction (yellow arrows) in groups 3 and 4 compared with groups 1 and 2. Remarkable alleviation in renal tubular damage in group 5 compared to groups 3 and 4. Also note the thickened basal membrane (green arrowheads) and dilated Bowman's capsules (black asterisks) and fibrotic changes in interstitial areas of kidney (blue asterisks) in groups 3 and 4 compared with other groups by 24-week after CKD induction. **L**) * vs. other groups, p < 0.0001 (by one-way ANOVA). Symbols (*, †, ‡, §) indicate significance (at 0.05 level by Bonferroni multiple comparison post hoc test). Scale bars in right lower corner represent 50 μm in **A to E** (n = 10 in each group).

Besides, to determine whether rosuvastatin therapy protected the integrity of renal-tubular architecture from NP + AD damage, PAS staining for identification of renal tubular brush border was performed(Figure 3). As expected, the number of intact renal-tubular brush border was notably higher in group 5 than in groups 3 and 4, suggesting that rosuvastatin therapy significantly improved the integrity of renal-tubular architecture from AD or NP + AD injury (Figure 3).

### Protein expressions of inflammatory, ROS, oxidative-stress biomarkers, and anti-oxidative mediators in renal parenchymal tissues

**Figure 4:**
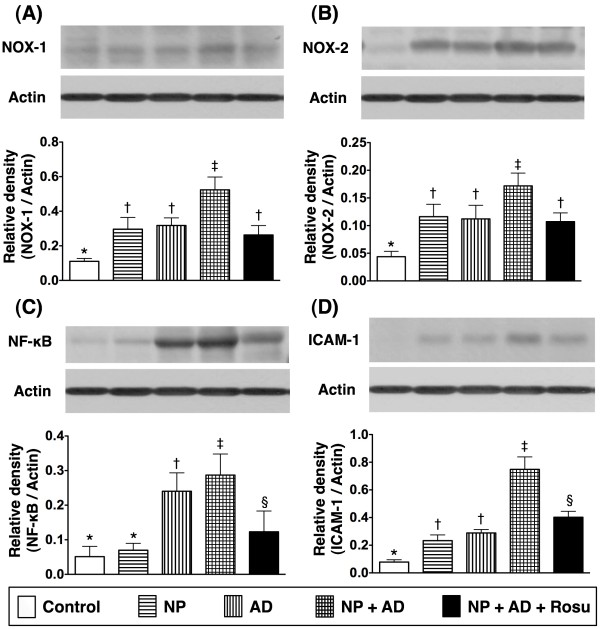
**Changes in protein expressions of inflammatory markers in kidney 24 weeks after CKD induction A & B).** Remarkably increased protein expressions of NADPH oxidase (NOX)-1 and NOX-2 in groups 2 and 3 than in group 1. Notably higher expressions in group 4 than in groups 1, 2, and 3, but significantly decreased in group 5 compared with group 4. For NOX-1 and NOX-2, *p < 0.001 between indicated groups. **C**) Notably elevated protein expression of nuclear factor (NF)-κB in group 3 than in groups 1 and 2. Further increase in group 4 compared with in groups 1, 2, and 3, but significantly lower in group 5 than in group 4. *p < 0.002 between indicated groups. **D**) Significantly higher protein expression of inter-cellular adhesion molecule (ICAM)-1 in groups 2 and 3 than in group 1. Further remarkable increase in group 4 compared with groups 1, 2, and 3, but notably lower in group 5 than in group 4. *p < 0.001 between indicated groups. All statistical analyses using one-way ANOVA, followed by Bonferroni multiple comparison post hoc test. Symbols (*, †, ‡, §) indicate significance (at 0.05 level) (n = 10 in each group).

**Figure 5:**
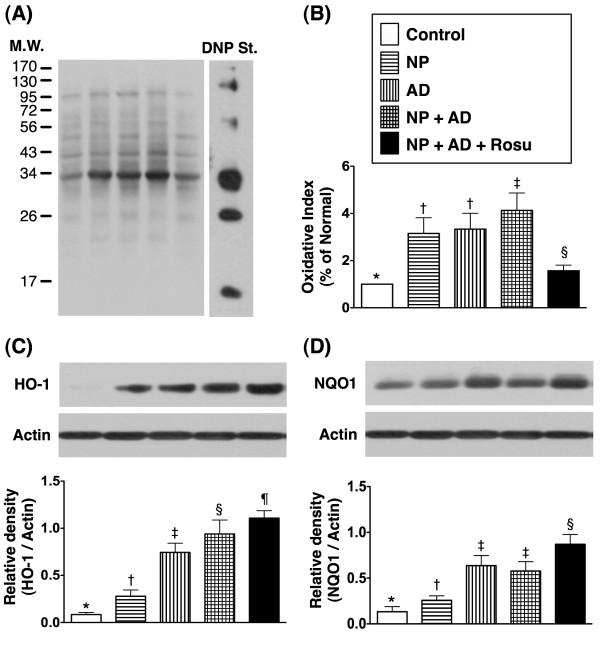
**Changes in protein expressions of oxidative stress and anti-oxidant markers in kidney at 24-week after CKD induction A & B).** Significantly increased oxidized protein expression in groups 2 and 3. Further increase in group 4 compared to groups 1, 2 and 3, but remarkably reduced in group 5 compared with group 4. * vs. other groups, p < 0.001. **C**) Notably increased protein expression of heme oxygenase (HO)-1 in groups 3 and 4 compared with that in groups 1 and 2. Significantly increased expression in group 2 than in group 1, and highest in group 5 compared with that in other groups. * vs. other groups, p < 0.0001. **D**) Notably higher protein expression of NAD(P)H quinone oxidoreductase (NQO) 1 in groups 3 and 4 than in groups 1 and 2, and highest in groups 5 compared with that in other groups. * vs. other groups, p < 0.0001. All statistical analyses using one-way ANOVA, followed by Bonferroni multiple comparison post hoc test. Symbols (*, †, ‡, §, ¶) indicate significance (at 0.05 level). (n = 10 in each group).

The protein expressions of NADPH oxidase (NOX)-1 (Figure 4A) and NOX-2 (Figure 4B), indicators of ROS generation, were notably higher in groups 2 and 3 than in group 1, and more markedly increased in group 4 than in groups 1 to 3, but remarkably reduced in group 5 as compared with group 4 at the end of the study period. Additionally, the protein expressions of NF-κB (Figure 4C), an indicator of inflammation, was notably higher in group 3 than in groups 1 and 2, more remarkably higher in group 4 compared with those in groups 1 to 3, but it showed significantly reduced in group 5 as compared with group 4. Moreover, the protein expression of ICAM-1 (Figure 4D), another indicator of inflammatory biomarker, was significantly enhanced in groups 2 and 3 than in group 1, further enhanced in group 4 than in group in groups 1, 2 and 3, but this parameter was notably reduced in group 5 than in group 4 at the end of the study period. Furthermore, the protein expression of oxidative stress, i.e. protein carbonyls (Figure 5A), was increased several folds in groups 2 and 3 as compared with group 1 and further increased in group 4 than in groups 1 to 3, but it was remarkably lower in group 5 than in groups 2 to 4. These findings suggest that AD-NP-induced CKD is through inflammation and ROS generation that could be suppressed by rosuvastatin therapy.

In contrast, the protein expressions of HO-1 (Figure 5C) and NQO1 (Figure 5D), two anti-oxidative biomarkers, were remarkably higher in groups 3 and 4 than in groups 1 and 2 and further higher in group 5 than in groups 3 and 4. These findings suggest that anti-oxidant generation was elicited in response to AD- and AD-NP-induced CKD and this protective mechanism was further up-regulated by rosuvastatin therapy in this setting of CKD.

### The mRNA and protein expressions of apoptotic and anti-apoptotic biomarkers

**Figure 6:**
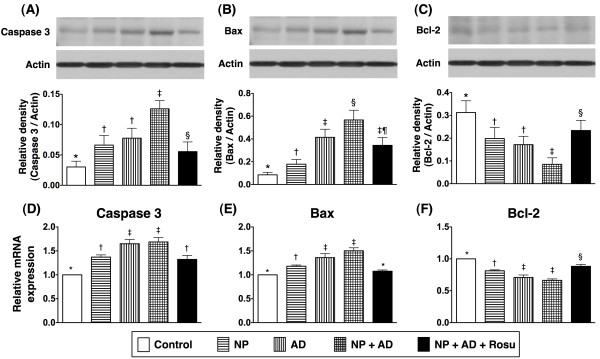
**The protein and mRNA expressions of apoptotic and anti-apoptotic biomarkers in kidney at 24-week after CKD induction A).** Significantly higher protein expression of caspase 3 in groups 2 and 3 than in group 1. Further increase in group 4 than in groups 1, 2, and 3, but drastically reduced in group 5 compared with that in group 4. * vs. other groups, p < 0.0001. **B**) Significantly elevated protein expression of Bax in group 2 than in group 1. Further increase in group 3 than in group 2, and highest in group 4. Notable reduction in group 5 than in group 4. * vs. other groups, p < 0.0001. **C)** Remarkably suppressed protein expression of Bcl-2 in group 4 than in groups 2 and 3. Markedly reduced expression in groups 2, 3, and 4 than in groups 1 and 5. Note significant reduction in group 5 compared with group 1. * vs. other groups, p < 0.0001. **D**) Significantly higher mRNA expression of caspase 3 in group 2 than in group 1. Notably increased expression in groups 3 and 4 than in group 2, but significantly reduced in group 5 compared with groups 3 and 4. * vs. other groups, p < 0.001. **E**) Markedly increased mRNA expression of Bax in group 2 than in group 1, further increased in groups 3 and 4 than in groups 1 and 2, but significantly reduced in group 5 than in groups 2 to 4. * vs. other groups, p < 0.0001. **F**) Remarkably lower mRNA expression of Bcl-2 in groups 3 and 4 than in group 2, and notably lower in group 2 than in groups 1 and 5. Significantly lower expression in group 5 than in group 1. * vs. other groups, p < 0.001. All statistical analyses using one-way ANOVA, followed by Bonferroni multiple comparison post hoc test. Symbols (*, †, ‡, §) indicate significance (at 0.05 level) (n = 10 in each group).

The protein expressions of caspase 3 (Figure 6A) and Bax (Figure 6B), two apoptotic biomarkers, were notably increased in groups 2 and 3 than in group 1, remarkably elevated in group 4 compared with groups 1 to 3, but they showed marked reductions in group 5 compared to group 4. Conversely, the protein expression of Bcl-2 (Figure 6C), an index of anti-apoptosis, was notably reduced in groups 2 and 3 compared with group 1, more markedly reduced in group 4 than in groups 2 and 3. However, it was significantly increased in group 5 compared to groups 2, 3, and 4.

Similarly, the mRNA expressions of caspase 3 (Figure 6D) and Bax (Figure 6E) were notably higher in group 2 than in group 1, further higher in groups 3 and 4 than in groups 1 and 2, but they were markedly lower in group 5 than in groups 3 and 4. In contrast, the mRNA expression of Bcl-2 (Figure 6F) was remarkably lower in groups 2 to 4 than in group 1, but it was significantly higher in group 5 than in groups 2 to 4. These findings suggest that the apoptotic signaling pathway, which is also involved in AD-NP-induced CKD, could be reversed by rosuvastatin therapy.

### Changes in mRNA expression of vasoactive, inflammatory, and anti-oxidative mediators in renal parenchyma after CKD induction

**Figure 7:**
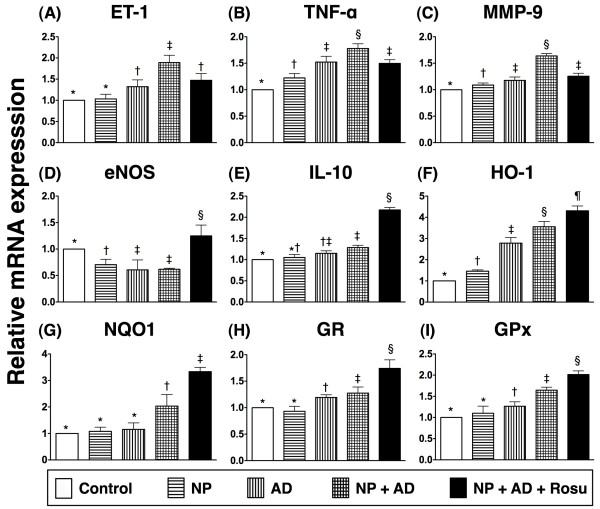
**Changes in mRNA expressions in kidney at 24-week after CKD induction A).** Significantly higher endothelin (ET)-1 expression in groups 2 and 3 than in group 1. Further increase in group 4 compared with groups 1 to 3, but significantly lower in group 5 than in group 4. **B**) Notably elevated tumor necrosis factor (TNF)-α expression in group 2 than in group 1, higher in group 3 than in groups 1 and 2, and highest in group 4. **C**) Significantly increased matrix metalloproteinase (MMP)-9 expression in groups 2 and 3 than in group 1, and highest in group 4. **D**) Remarkably lower endothelial nitric oxide synthase (eNOS) expression in groups 2, 3, and 4 than in group 1, and highest in group 5. **E**) Significantly higher interleukin (IL)-10 expression in group 4 than in groups 1 and 2, and highest in group 5. **F**) Significantly higher HO-1 expression in group 3 than in groups 1 and 2, further increased in group 4 compared with groups 1 to 3, and highest in group 5. **G & H**) Notably increased expressions of NQO 1 and glutathione reductase (GR) in group 4 than in groups 1 to 3, and highest in group 5. **I**) Significantly increased glutathione peroxidase (GPx) expression in group 3 than in groups 1 and 2, further increased in group 4 than in groups 1 to 3, and highest in group 5. All statistical analyses using by one-way ANOVA, followed by Bonferroni multiple comparison post hoc test. Symbols (*, †, ‡, §, ¶) indicate significance (at 0.05 level) (n = 10 in each group).

The mRNA expression of endothelin (ET)-1 (Figure 7A), an index of endothelial damage/vasoconstriction, was notably higher in group 3 than in groups 1 and 2, further markedly higher in group 4 than in groups 1 to 3. However, it was remarkably lower in group 5 than in group 4. The mRNA expressions of tumor necrosis factor (TNF)-α (Figure 7B) and matrix metalloproteinase (MMP)-9 (Figure 7C), two indicators of inflammation, were remarkably higher in group 3 than in groups 1 and 2, further higher in group 4 than in groups 1 to 3, but they were notably reduced in group 5 compared to group 4. On the other hand, the mRNA expressions of endothelial nitric oxide synthase (eNOS) (Figure 7D) an anti-inflammatory index, was notably lower in groups 2 and 3 than in group 1, and significantly reduced in group 4 compared with group 1. However, this biomarker was substantially increased in group 5 compared with groups 2 to 4. The mRNA expression of interleukin (IL)-10 (Figure 7E), another anti-inflammatory index, was significantly increased in group 4 than in groups 1 and 2, and it showed further increased in group 5 as compared with groups 1 to 4. Additionally, the mRNA expressions of HO-1 (Figure 7F), NQO1 (Figure 7G), glutathione reductase (GR) (Figure 7H), and glutathione peroxidase (GPx) (Figure 7I), four anti-oxidative indicators, were significantly higher in group 4 than in groups 1 to 3, and more markedly increased in group 5 than in group 4. These findings suggest that anti-inflammatory and anti-oxidative responses after induction of CKD with AD & NP and enhancement of these responses following rosuvastatin therapy.

### Findings from IF and IHC staining

**Figure 8:**
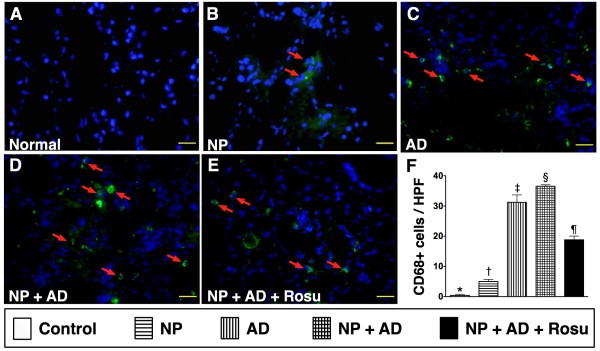
**Immunofluorescent (IF) staining for renal expressions of inflammatory markers at 24-week after CKD induction A to E).** Identification of macrophage (CD68+ cells) (red arrows) accumulation in kidney parenchyma of each group of rats using IF staining, showing significantly higher number of macrophage in group 2 than in group 1. Further increase noted in group 3 than in groups 1 and 2, and substantially higher in group 4 than in groups 1 to 3, but markedly lower in group 5 compared with groups 3 and 4. **F**) * vs. other groups, p < 0.0001. Nuclei (blue color) stained with 4’,6-diamidino-2-phenylindole (DAPI) as counter staining. All pictures taken under high-power field (HPF) 400x. Scale bars in right lower corner represent 50 μm. All statistical analyses using one-way ANOVA, followed by Bonferroni multiple comparison post hoc test. Symbols (*, †, ‡, §, ¶) indicate significance (at 0.05 level) (n = 10).

The IF staining for CD68-positive cell (Figure 8), a surface marker of macrophage in kidney tissue, was found to be remarkably increased in groups 3 and 4 than in groups 1 and 2, but it showed notably reduced in group 5 than in groups 3 and 4. These findings suggest that rosuvastatin treatment offered an anti-inflammatory effect.

**Figure 9:**
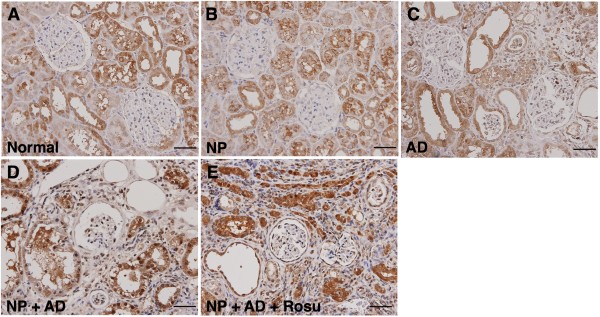
**Immunohistochemical (IHC) staining for renal expressions of anti-oxidant markers at 24-week after CKD induction A to E).** Identification of GR-positive cells accumulation in kidney parenchyma of each group using IHC staining (200x). Note remarkably increased expression in group 4 than in groups 1 to 3, and highest in group 5. Scale bars in right lower corner represent 50 μm. (n = 10).

The IHC staining (Figure 9) revealed that the expression of GR, an anti-oxidative enzyme, was remarkably higher in group 4 than in groups 1 to 3, and highest in group 5. These findings, together with results from RT-PCR (Figure 7H), further suggest that anti-oxidative response was elicited by rosuvastatin treatment contributed to anti-oxidative effects after AD & NP-induced CKD in this study.

## Discussion

This study, which used a rodent model to investigate the therapeutic impact of rosuvastatin therapy on AD-NP-induced CKD, provided several striking implications. First, the major contributors to AD-NP-induced CKD were found to be inflammatory reaction, ROS generation, and cellular apoptosis. Second, rosuvastatin treatment significantly preserved architectural integrity of renal parenchyma and attenuated the deterioration of renal function after AD-NP-induced kidney damage. Third, rosuvastatin therapy consistently initiated anti-inflammatory, anti-apoptotic, and anti-oxidative effects.

### Aggravation of AD-Induced renal dysfunction by NP

Although AD-induced CKD animal model has been validated in previous studies [27-29], the authors of those studies used a relatively higher dose of AD (i.e. 0.75%) compared with that used in the present study (i.e. 0.25%). Our results, therefore, demonstrated that AD successfully induced CKD in a rodent model even at one-third of the reported dose. Furthermore, the novel finding of the current study is that NP feeding aggravated the deterioration of AD-induced renal function impairment. This finding implicates that not only is NP an endocrine-disrupting organic compound in the environment [25], but it also participates in deteriorating the mammalian renal function in subjects with pre-existing chronic renal disease.

One important finding in the present study is that both gross anatomical and histological findings showed irregular appearance and crystal depositions in kidneys after AD treatment. Consistently, previous studies [27-29] have also shown similar findings. Besides, another finding of interest in the current study is that despite the lack of significant difference in renal functions (i.e. serum BUN and creatinine levels) between the normal controls and NP-fed animals by the end of study period, immense differences in renal parenchyma including the histological pictures, mRNA, and protein expressions of various inflammation- and apoptosis-related molecules were noted between the two groups. Our findings, therefore, may suggest that NP, an environmental organic compound [25], may play an essential role in aggravating mammalian CKD on chronic exposure.

### The possible mechanisms of AD-NP-induced CKD

Interestingly, although AD-induced CKD in rodents is a well-established model [27-29], the mechanistic basis of AD-induced CKD has seldom been investigated [27-29]. Additionally, the mechanism of AD-NP-induced CKD in the rat model has not been reported. Another important finding in the present study is that the expressions of inflammatory markers including CD68-positive cells, TNF-1α, MMP-9, ICAM-1, and NF-κB were notably higher in NP-treated animals (group 2), and further increased in the AD-treated group (group 3), and highest in the AD-NP-treated group (group 4) compared with those in the normal controls (group 1). The principal findings of the present study are that ROS production (NOX-1, NOX-2), oxidized protein expression (protein carbonyls), and endothelial damage index (ET-1 gene expression) were notably increased in group 2, further increased in group 3, and substantially elevated in group 4 compared with group 1. Numerous previous studies have shown that CKD elicits rigorous inflammatory reaction, oxidative stress, and the generation of ROS [1,18,19,30-33], which, in turn, cause further damage to renal parenchyma and destroy the architectural integrity of the kidney. In this way, our findings, in addition to reinforcing those of previous studies [1,18,19,30-33], further showed that AD, NP, and combined AD-NP treatment caused different degrees of renal damage in a rodent model of CKD.

Of importance is that, as compared with group 4 animals, the expressions of these inflammatory and oxidative biomarkers at both gene and protein levels were significantly suppressed in group 5 animals following rosuvastatin administration. Moreover, the generations of anti-oxidants at IHC stain (GR-positive cells), protein (HO-1 and NQO1) and mRNA (HO-1, NQO1, GR, and GPx) levels were also markedly enhanced in group 5 animals compared with those in group 4. Besides, at both protein and mRNA levels, the apoptotic biomarkers (caspase 3 and Bax) were markedly reduced, whereas the anti-apoptotic biomarker (Bcl-2) and anti-inflammatory biomarkers at gene level (IL-10, eNOS) were significantly enhanced in group 5 animals compared with those in group 4. These results, at least in part, may suggest the involvement of the apoptotic signaling pathway in CKD. Our findings further support those of previous studies showing that cellular apoptosis/death is always present in CKD [39,40].

A body of evidence has demonstrated that statin has distinctive properties of being anti-inflammatory [33], inhibiting ROS production [41,42] and reducing oxidant/free radical generations [43,44]. Using an animal model of AD-NP-induced CKD, the results of the present study also revealed that rosuvastatin also possesses all of the above-mentioned distinctive properties [33,41-43]. Our findings, therefore, in addition to corroborating those of previous studies [33,41-44], suggest the potential benefit of early statin use in the setting of CKD.

### Study limitations

This study, however, has its limitations. First, although multifaceted signaling pathways were found to be involved in this AD-NP-induced CKD animal model, we remain uncertain how many signaling pathways exactly contribute to our observations. Second, the results of this study did not specify the most important mechanism involved in AD-NP-induced CKD. Third, we remain uncertain for why a bit different association between antioxidants and detoxifying enzymes and oxidative-stress biomarkers were found in the current study. Interestingly, a similar result has also been found recently in a rat animal model of acute kidney ischemia-reperfusion injury with stem cell therapy [36]. We suggest that the findings in the current study may implicate that inherent anti-oxidant system can be initiated by CKD in response to NP, AD, and AD + NP stress and that this effect could be further enhanced by rosuvastatin therapy.

## Conclusions

The present study revealed that chronic NP treatment aggravated AD-induced CKD in a rodent model. The underlying mechanisms include augmentation of inflammatory reaction, generation of ROS, and enhancement of cellular apoptosis which were found to be suppressed by rosuvastatin therapy.

## Competing interests

The authors declare that they have no competing interests.

## Authors’ contributions

CHY, CKS, SL, YTC, and HKY designed the experiment, drafted and performed animal experiments. YCL, LTC, YLC, THT, YHK, PLS were responsible for the laboratory assay and troubleshooting. CYH, CGW, SL and HKY participated in refinement of experiment protocol and coordination and helped in drafting the manuscript. All authors read and approved the final manuscript.
